# Development and Application of Quantitative Detection Method for Viral Hemorrhagic Septicemia Virus (VHSV) Genogroup IVa

**DOI:** 10.3390/v6052204

**Published:** 2014-05-23

**Authors:** Jong-Oh Kim, Wi-Sik Kim, Si-Woo Kim, Hyun-Ja Han, Jin Woo Kim, Myoung Ae Park, Myung-Joo Oh

**Affiliations:** 1 Department of Aqualife Medicine, Chonnam National University, Yeosu 550-749, Korea; E-Mails: jongoh.kim77@gmail.com (J.-O.K.); wisky@chonnam.ac.kr (W.-S.K.); kimman2309@naver.com (S.-W.K.); 2 Aquatic Life Disease Control Division, National Fisheries Research and Development Institute, Busan 619-902, Korea; E-Mails: hjhan77@korea.kr (H.-J.H.); jwkim123@korea.kr (J.W.K.); mapark@korea.kr (M.A.P.)

**Keywords:** viral hemorrhagic septicemia virus (VHSV), quantitative detection, real-time PCR, diagnostic, olive flounder

## Abstract

Viral hemorrhagic septicemia virus (VHSV) is a problematic pathogen in olive flounder (*Paralichthys olivaceus*) aquaculture farms in Korea. Thus, it is necessary to develop a rapid and accurate diagnostic method to detect this virus. We developed a quantitative RT-PCR (qRT-PCR) method based on the nucleocapsid (N) gene sequence of Korean VHSV isolate (Genogroup IVa). The slope and R^2^ values of the primer set developed in this study were −0.2928 (96% efficiency) and 0.9979, respectively. Its comparison with viral infectivity calculated by traditional quantifying method (TCID_50_) showed a similar pattern of kinetic changes *in vitro* and *in vivo*. The qRT-PCR method reduced detection time compared to that of TCID_50_, making it a very useful tool for VHSV diagnosis.

## 1. Introduction

In recent years, fish consumption has increased worldwide because it is a healthy source of nutrients that is rich in proteins, minerals, and essential fatty acids. As olive flounder (*Paralichthys olivaceus*) is one of the preferred food sources in Korea, there has been an increase in the number of olive flounder farms. However, many farms have suffered substantial financial loss due to the occurrence of viral hemorrhagic disease (VHS) [1,2,3].

VHS virus (VHSV) is the etiological agent of VHS, which is one of the most serious viral diseases affecting farmed rainbow trout (*Oncorhynchus mykiss*) in European countries and olive flounder (*Paralichthys olivaceus*) in Far East Asia [2,4,5,6,7]. VHSV is a *Novirhabdovirus* RNA virus belonging to the family *Rhabdoviridae* and has a negative sense single-stranded RNA genome with approximately 11.1 kb containing six genes, nucleocapsid protein (N), phosphoprotein (P), matrix protein (M), glycoprotein (G), non-virion protein (NV) and polymerase (L) genes, in the order 3’-N-P-M-G-NV-L-5’ [8]. Based on phylogenetic analyses with nucleotide sequences of N and G genes, worldwide isolates of VHSV were classified into four genotypes. Genotype I includes a wide range of viruses from freshwater and marine species in Europe, genotype II is composed of marine isolates from the Baltic Sea, genotype III includes isolates from the British Isles and Scottish waters, and genotype IV contains isolates from Korea, Japan, and the Pacific Coast and Great Lakes regions of North America [1,2,3,4,9,10,11,12,13,14,15,16]. Moreover, the Asian isolates (genotype IVa) are phylogenetically distinguishable from North American isolates [3,14]. Thus, the Far East Asian VHSV isolates could be an indigenous virus in Korean and Japanese coastal areas. Recently, Kim *et al.* analyzed complete genome sequence of VHSV isolated from olive flounder and confirmed it was included in a genotype IVa by phylogenetic study [17].

The World Organization for Animal Health Office International des Epizooties (OIE) has determined VHSV to be a problematic disease and has attempted to manage it [18] by providing an effective VHSV detection method such as the polymerase chain reaction (PCR), antibody-based assays and so on; however, it is not a quantitative assay. Therefore, additional cell culture-based experiments, *i.e.*, plaque assay or 50% tissue culture infectivity dose (TCID_50_) are necessary to quantify the virus, but it takes approximately 1–2 weeks to obtain results. Thus, it is important to develop detection methods to diagnose VHS within a short time. Recently, the use of quantitative RT-PCR (qRT-PCR) in the field of virus disease diagnosis has increased because it is rapid, sensitive and has high specificity [19]. The qRT-PCR technique allows many samples can be analyzed simultaneously within a short time and is providing great impulse for the upgrading of previous diagnostic assays. Recently, although some genotype-specific quantitative detection methods for VHSV have been published [20,21], there is no reported method developed using genotype IVa.

Thus, in this study, we newly developed a quantitative detection method for VHSV genotype IVa using Korean isolate and compared it with the conventional bio-assay method (TCID_50_) using *in vitro* and *in vivo* samples.

## 2. Results and Discussion

### 2.1. Plasmid Construction for Standard and Primer Efficiency

**Figure 1:**
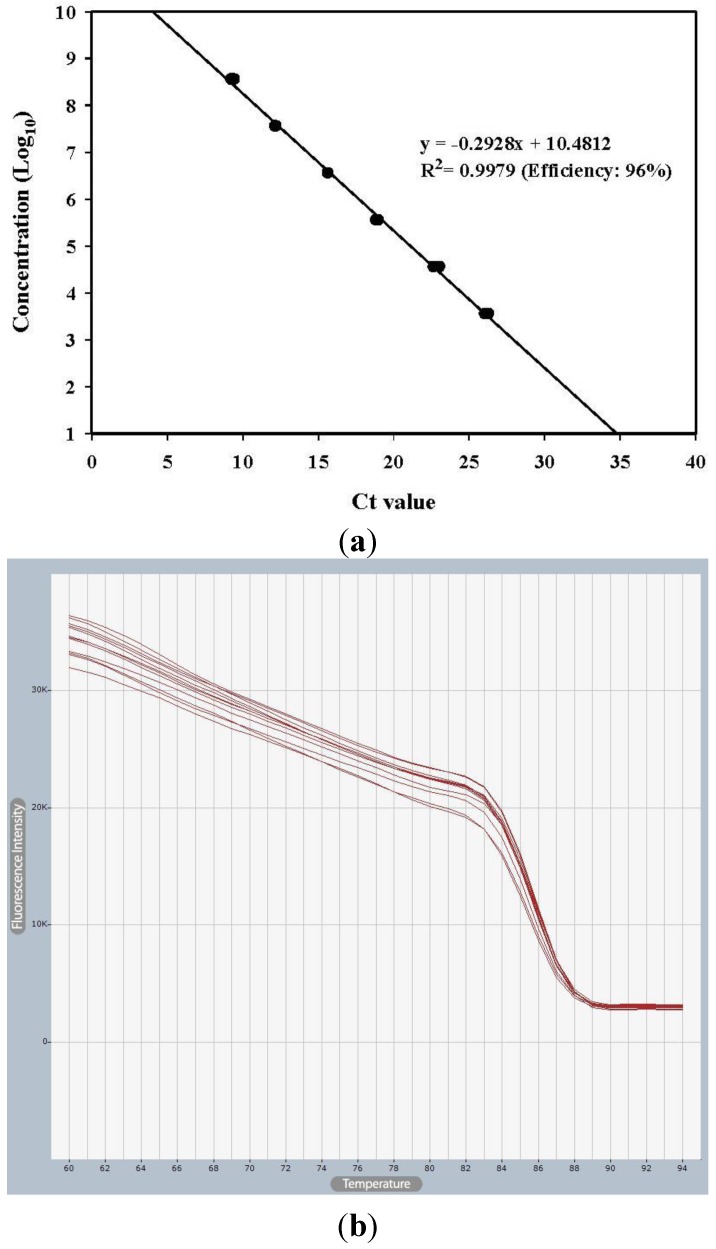
qRT-PCR standard curve (**a**) and melting curve (**b**). The plasmid DNA harboring viral hemorrhagic septicemia virus (VHSV) N gene was diluted by 10-fold and amplified in duplicate by quantitative PCR reaction. Standard curve for this N gene primers; slope = −0.2928, R^2^ = 0.9979.

It was amplified with an open reading frame (ORF) of nucleoprotein (N) gene from VHSV FYeosu05 isolate and cloned. The serial 10-fold dilutions of the cloned plasmid were amplified in duplicate by qRT-PCR to determine the sensitivity of the qRT-PCR assay. The slope and R^2^ values of the primer set used in this study were −0.2928 (96% efficiency) and 0.9979, respectively (Figure 1). The primer set showed an equivalent efficiency and satisfactory coefficient of determination (R^2^) values compared to other studies [21,22,23].

### 2.2. Comparison of qRT-PCR and TCID_50_ Method (In Vitro)

**Figure 2:**
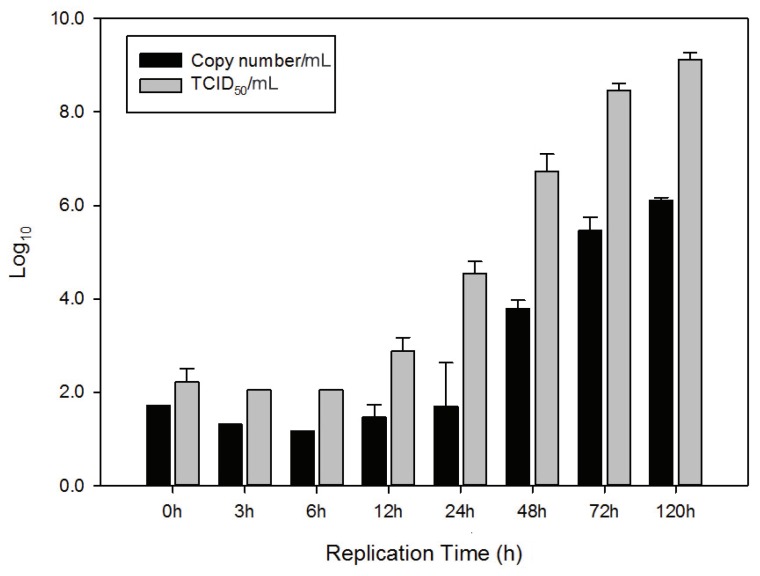
Comparison of VHSV titer (TCID_50_/mL) and copy number (*in vitro*). Black bar indicates log values of the VHSV copy number and gray bar indicates log values of VHSV titer (TCID_50_/mL).

Figure 2 reveals both replication curves in VHSV infectivity by the TCID_50_/mL value (gray bar) and viral copy numbers calculated by qRT-PCR (black bar). Infectivity was lower than 10^3.1^ TCID_50_/mL until 12 h after infection and then virus replication increased rapidly between 12 h (10^3.1^ TCID_50_/mL) and 3 days (10^8.6^ TCID_50_/mL). About 10^9.3^ TCID_50_/mL titers were measured at 5 days. Consequently, we considered that the viruses were released into the culture medium after 12 h. Similarly, the viral copy number remained almost unchanged until 24 h (about 10^1.5^ copies/mL) and then gradually increased until 72 h. About 10^6.1^ copies/mL were maintained until 5 days. This is the first comparison study of the change in VHSV replication kinetics and its N gene copy numbers *in vitro*. It showed similar change patterns. Pierce *et al.* revealed that quantitation of VHSV molecules in the dilution test series followed a linear relationship to results from plaque assays [24].

### 2.3. Comparison of qRT-PCR and TCID_50_ Method with VHSV Challenged Olive Flounder (In Vivo)

**Figure 3:**
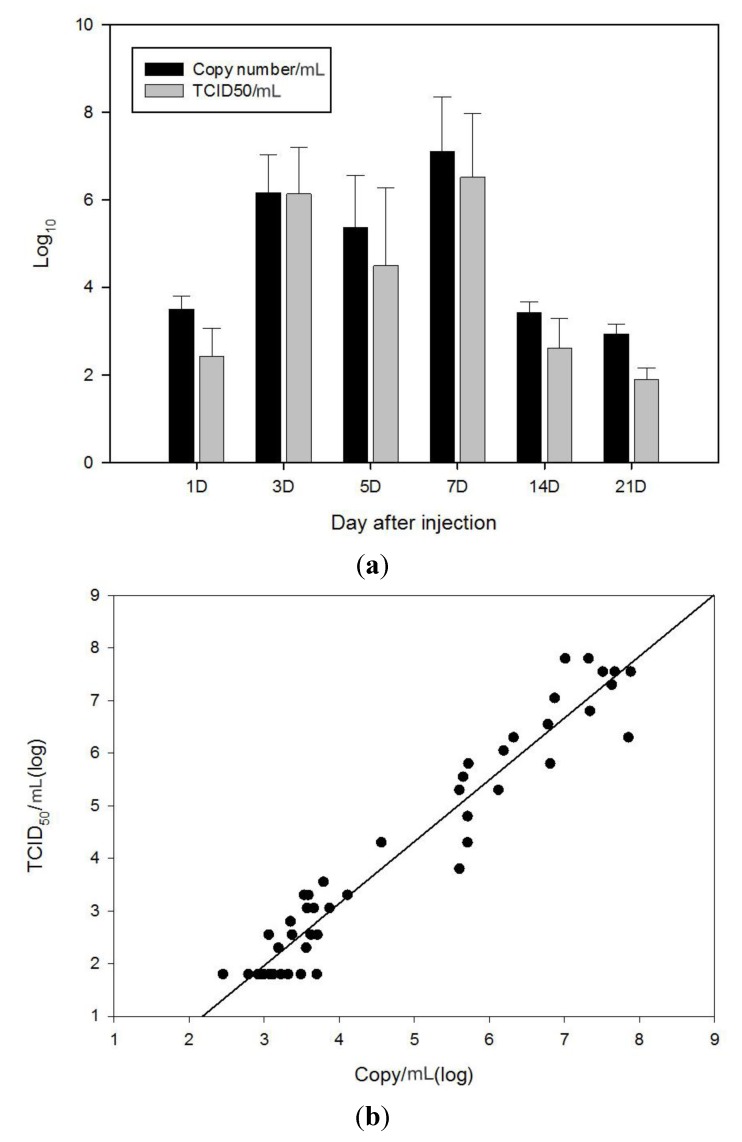
Comparison of VHSV titer (TCID_50_/mL) and copy number (*in vivo*). (**a**) Black bar indicates average log values of the VHSV copy number and gray bar indicates average log values of VHSV titer (TCID_50_/mL); (**b**) The copy numbers of VHSV determined from qRT-PCR *versus* those from VHSV titer (TCID_50_/mL) for individual samples.

VHSV infection caused mortality in the olive flounder. Most of the dead fish exhibited ascites, congested liver and enlarged spleen and kidney. The challenged fish died during day 6–22 after VHSV infection and cumulative mortality was estimated as 63.8% (Figure S1). A total of 48 fish samples (eight fish from each sampling time) were used to determine average viral copy numbers/mL and TCID_50_/mL and these results are in Figure 3a. VHSV was detectable in five out of eight fish on the 1st day after injection (D.A.I.) with 10^2.43^ TCID_50_/mL of the average infectivity titer, but VHSV rapidly multiplied on the 3rd D.A.I. The VHSV titers on the 3rd, 5th, 7th D.A.I. were 10^6.14^, 10^4.55^, 10^6.52^ TCID_50_/mL, respectively, and then VHSV reduced to 10^2.61^ TCID_50_/mL on the 14th D.A.I. VHSV was detected only in one fish on the 21st D.A.I. These results demonstrate that VHSV multiplied very quickly at 3–5 D.A.I. before the fish began to die. Similarly, the viral copy number at the 1st D.A.I. was 10^3.5^ copies/mL (Ct 34.1) but it went up to 10^6.17^ copies/mL (Ct 24.97) at the 3rd D.A.I. The VHSV copy numbers on the 5th and 7th D.A.I. were 10^5.37^, 10^7.10^ copies/mL (Ct 27.69, 21.79), respectively, and then it decreased to 10^3.43^ copies/mL (Ct 34.34) at 14th D.A.I. On the 21st D.A.I. it recorded 10^2.94^ copies/mL (Ct 36.0). Figure 3b represents the copy numbers of VHSV *versus* the titers (TCID_50_/mL) for individual samples. From these results presented herein, the changes of VHSV titer were comparable to that of the copy numbers. Interestingly, the VHSV titers were rather lower than copy numbers in *in vivo* results while the VHSV titers were higher than copy numbers in *in vitro* results (Figure 2). It might be that the *in vivo* tissue sample contained all forms of the viral genome, and not only assembled viral genomes in complete virion but also extra viral genomes such as replication intermediates and immature viruses. Furthermore, the *in vivo* sample contained lots of inhibitors like proteases which could affect virus activity. These can make a difference between *in vitro* and *in vivo* results. It is necessary to conduct extra studies with more field samples and find accurate reasons as to what causes the differences between *in vitro* and *in vivo* results to better understand the efficiency of qRT-PCR method and its relationship with the TCID_50_ method. Hope *et al.* compared the qRT-PCR assay and cell culture protocols using wild fish samples [22]. From this study they also explained that the qRT-PCR was as accurate as cell culture identification but far more sensitive with respect to the detection of VHSV infection.

## 3. Experimental Section

### 3.1. Virus and Cells

The VHSV (FYeosu05) used in this study was isolated in 2005 from Yeosu, Korea [2] and propagated in the fathead minnow (FHM) cell line. FHM cells were grown at 20 °C in Leibovitz L-15 medium (Sigma Aldrich, St. Louis, MO, USA) with 10% fetal bovine serum (Gibco, Gland Island, NY, USA), 150 U/mL penicillin G, and 100 µg/mL streptomycin. VHSV was inoculated on a confluent FHM cell monolayer and incubated at 15 °C to replicate the virus. Viral samples were aliquot in small volumes and stored at −80 °C until use.

### 3.2. Viral RNA Extraction and cDNA Synthesis

Viral RNA was extracted with miRNA extraction kit (Qiagen, Hilden, Germany) and cDNA was synthesized using M-MLV Reverse Transcriptase (Bioneer, Daejeon, Korea) following the manufacturer’s protocols. For cDNA synthesis, the mixture of extracted RNA and 20 pmole of qVN_11F primer (5’-GAATCCGTGCAGCTTTTTCAGG-3’) was incubated at 65 °C for 10 min, and immediately cooled on ice. Secondly, the mixture mixed with 4 µL of 5× MMLV Reverse Transcriptase reaction buffer, 2 µL of 100 mM DTT, 1 mM dNTP (each), 0.5 µL of RNase inhibitor (40 U/µL), 0.5 µL of MMLV RTase (10 U/µL) and incubated for 60 min at 37 °C. Finally, the mixture was incubated for 5 min at 85 °C to inactivate RTase. The synthesized cDNA was stored at –20 °C for quantitative PCR and further use.

### 3.3. Gene Cloning

The nucleoprotein gene open reading frame (ORF) of VHSV was cloned into the pCR2.1-Topo vector (Invitrogen, Carlsbad, CA, USA) to prepare a standard curve. The VHSV genes were amplified with the ORF primer set (VN_ORF_for, ATGGAAGGGGGAATCCGTGC; VN_ORF_rev, TTAATCAGAGTCCCCTGGGTAGTCGT) and the PCR product was eluted with a GeneAll Expin Gel SV kit (GeneAll, Seoul, Korea). The purified PCR products were ligated with the pCR2.1-Topo vector transformed to *Escherichia coli* Top10 strain. Plasmid DNA was extracted with an AccuPrep Plasmid Mini Extraction kit (Bioneer, Daejeon, Korea). DNA concentration was measured using a NanoDrop ND-1000 spectrophotometer (Thermo Scientific, Pittsburgh, PA, USA).

### 3.4. Quantitative RT-PCR (qRT-PCR)

A primer set (qVN_11F, GAATCCGTGCAGCTTTTTCAGG; qVN_rev_160R, CAAGTGCATCCACGATCACCTTC) used in this work were designed by Primer3Plus [25] based on the VHSV FYeosu05 genome sequence [18]. Primer efficiency was examined by the qRT-PCR with 10-fold diluted plasmid DNA. The qRT-PCR was carried out in an Exicycler 96 Real-Time Quantitative Thermal Block (Bioneer, Daejeon, Korea) using SYBR green mixture, AccuPower Greenstar qPCR Premix (Bioneer, Daejeon, Korea). The reaction conditions were set as stated in the manufacturer’s instructions. Briefly, a 10 min pre-denaturation cycle at 95 °C, 40 cycles of 20 s denaturation at 95 °C, and a 40 s extension at 58 °C were used. The specification of the qRT-PCR reaction was analyzed through melting curve analysis, and the baseline was determined automatically by the Exicycler Analysis Software (Bioneer, Daejeon, Korea).

### 3.5. Comparison qRT-PCR and TCID_50_ Method (In Vitro)

A 0.015 multiplicity of infection virus was inoculated into six 25 cm^2^ culture flasks containing FHM cells and 200 µL of the supernatant was sampled immediately after virus inoculation (0 h), at 3, 6, 12, 24 h, and at 2, 3, and 5 days from each of six culture flasks to compare the TCID_50_ method and the qRT-PCR method. Three samples were used for virus titration by the TCID_50_ method and the other three samples were used for qRT-PCR. FHM cells (10^5^ cells/well) were cultured in 96-well plates, and 50 µL of 10-fold diluted virus (10^−1^–10^−9^) was inoculated onto the 96-well plates. TCID_50_ value was calculated 14 days after inoculation using the Reed and Muench method [26]. All samples were statistically analyzed and all the data were represented as the means ± the standard error.

### 3.6. Comparison of qRT-PCR and TCID_50_ Method with VHSV Challenged Olive Flounder (In Vivo)

Three hundred fish were cultivated in two aquaria (*n* = 150), separately. VHSV at dose of 10^5.8^ TCID_50_/100 µL/fish was injected intramuscularly into the fish in aquaria, while 100 µL of L-15 medium/fish was injected into fish in the remaining aquarium as a control. The challenged fish were reared and daily observed for three weeks (21 days). Spleen and kidney tissues of eight fish from each group were collected on the 1st, 3rd, 5th, 7th, 14th and 21st days for titration of VHSV and viral copy number. The obtained tissues were homogenized with nine volumes of DMEM medium and centrifuged at 5000× *g* for 20 min (4 °C), and then the supernatant was subdivided into small aliquots and stored at −80 °C until use.

## 4. Conclusions

Although many researchers commonly use the plaque assay or TCID_50_ method to quantify VHSV, it takes almost two weeks to obtain a final result. However, the qRT-PCR method developed in this study decreased experimental time shortly within a day*.* Moreover, the comparison of viral copy numbers with viral infectivity calculated by TCID_50_ shows a similar change pattern for 120 h after virus inoculation to FHM cells (*in vitro*). In addition, the changes of VHSV titer from 48 VHSV challenged fish were comparable to that of the copy numbers (*in vivo*). Thus, the qRT-PCR method developed here demonstrates great potential to quantitatively detect VHSV. It could be useful for rapid detection of VHSV in fish of aqua-farms and researches to understand the relationship between virus replication and occurrence of VHS.

## Supplementary Files

Supplementary File 1 Supplementary Information (PDF, 197 KB)
