# Supplementary material for: Development and Application of Quantitative Detection Method for Viral Hemorrhagic Septicemia Virus (VHSV) Genogroup IVa
Source: Viruses. 2014 May 23;6(5):2204–13. doi: 10.3390/v6052204 (PMC4036551; doi:10.3390/v6052204)

Supplementary Information

Figure S1. Cumulative mortality of flounder after VHSV challenge. The challenged fish died during day 6–22 after VHSV injection and cumulative mortality was estimated as 63.8%.

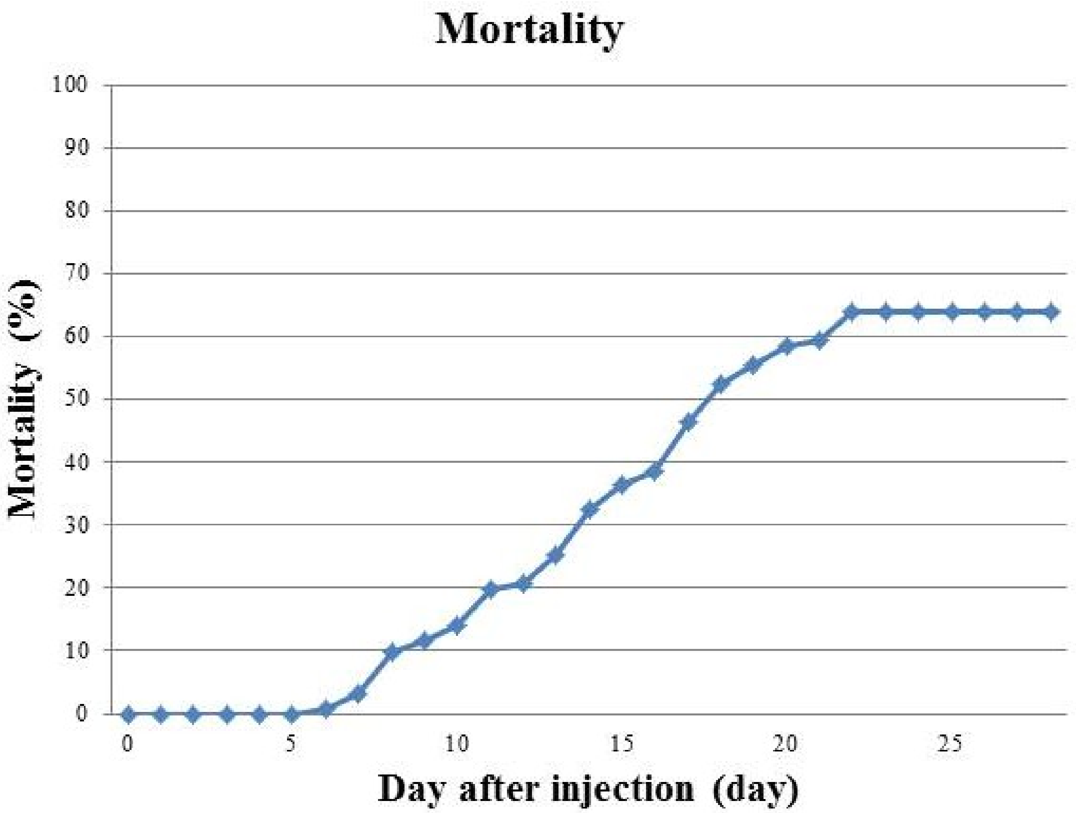

Supplement: Supplementary File 1 — Supplementary Information (PDF, 197 KB) [file viruses-06-02204-s001.pdf]
